# Integration Technology for Wafer-Level LiNbO_3_ Single-Crystal Thin Film on Silicon by Polyimide Adhesive Bonding and Chemical Mechanical Polishing

**DOI:** 10.3390/nano11102554

**Published:** 2021-09-29

**Authors:** Wenping Geng, Xiangyu Yang, Gang Xue, Wenhao Xu, Kaixi Bi, Linyu Mei, Le Zhang, Xiaojuan Hou, Xiujian Chou

**Affiliations:** 1 Science and Technology on Electronic Test and Measurement Laboratory, North University of China, Taiyuan 030051, China; s1806068@st.nuc.edu.cn (X.Y.); s1906061@st.nuc.edu.cn (G.X.); s202106135@st.nuc.edu.cn (W.X.); bikaixi@nuc.edu.cn (K.B.); zhangle@nuc.edu.cn (L.Z.); houxiaojuan@nuc.edu.cn (X.H.); chouxiujian@nuc.edu.cn (X.C.); 2 School of Mechanical Engineering, North University of China, Taiyuan 030051, China; mly81@163.com

**Keywords:** LiNbO_3_ single-crystal, polyimide material, silicon substrate, chemical mechanical polishing

## Abstract

An integration technology for wafer-level LiNbO_3_ single-crystal thin film on Si has been achieved. The optimized spin-coating speed of PI (polyimide) adhesive is 3500 rad/min. According to Fourier infrared analysis of the chemical state of the film baked under different conditions, a high-quality PI film that can be used for wafer-level bonding is obtained. A high bonding strength of 11.38 MPa is obtained by a tensile machine. The bonding interface is uniform, completed and non-porous. After the PI adhesive bonding process, the LiNbO_3_ single-crystal was lapped by chemical mechanical polishing. The thickness of the 100 mm diameter LiNbO_3_ can be decreased from 500 to 10 μm without generating serious cracks. A defect-free and tight bonding interface was confirmed by scanning electron microscopy. X-ray diffraction results show that the prepared LiNbO_3_ single-crystal thin film has a highly crystalline quality. Heterogeneous integration of LiNbO_3_ single-crystal thin film on Si is of great significance to the fabrication of MEMS devices for in-situ measurement of space-sensing signals.

## 1. Introduction

Lithium niobate (LiNbO_3_) crystal is widely used for sensors, detectors, electro-optical modulators, information storage and other micro-electromechanical systems (MEMS) devices because of its excellent piezoelectric, ferroelectric, acoustic-optical, non-linear optical effect and electromechanical coupling factors. In recent years, with the rapid development of space technology and micro-electromechanical systems, an increasing number of MEMS devices are used in the aerospace field [1,2,3,4]. The harsh environment such as low temperature and irradiation in outer space puts a huge test on the stability and lifetime of these MEMS devices [5,6,7,8,9]. LiNbO_3_ single-crystal has received extensive attention in the aerospace field owing to its low-temperature resistance and intrinsic radiation resistance [10,11,12]. Silicon (Si) is the most commonly used substrate material with excellent mechanical, electrical, and thermal properties, and its processing technology is compatible with the CMOS process [13,14,15]. Under the development trend of low power consumption, miniaturization and integration of space devices, it is vital to realize the integration of large-area, high-quality LiNbO_3_ single-crystal thin films on silicon.

There is a large lattice mismatch between LiNbO_3_ and Si, which makes the epitaxial growth of LiNbO_3_ thin films on Si substrates extremely challenging. The traditional growth methods of LiNbO_3_ thin films include RF magnetron sputtering, sol-gel, chemical vapour deposition and pulsed laser deposition [16,17,18,19,20]. It is difficult to prepare LiNbO_3_ thin films with low lattice defects and high stoichiometric ratios on silicon substrates through the above methods. Moreover, since LiNbO_3_ crystal is anisotropic, the physical properties depend on the crystal orientation, for example, the 128° Y-cut LiNbO_3_ has a higher electromechanical coupling factor [21]. However, it is impossible to grow these special orientation LiNbO_3_ films by the above thin film deposition methods. In recent years, the integration of LiNbO_3_ single-crystal thin films on substrates has been achieved through a crystal ion slicing (CIS) technique. For example, Pastureaud et al. fabricated YX-cut LiNbO_3_ single-crystal thin films on Si substrates by means of CIS [22]. Luo et al. fabricated LiNbO_3_ single-crystal thin films on LiNbO_3_ substrates by means of CIS for the fabrication of pyroelectric infrared detectors [23]. Wafer bonding is one of the key steps in the CIS process. The thermal mismatch between LiNbO_3_ and Si is serious, so the bonding must be performed at a low temperature. Bai et al. achieved the integration of the 128° Y-cut LiNbO_3_ single-crystal thin films on Si substrates via benzocyclobutene (BCB) adhesive [21]. But the LiNbO_3_/BCB/Si bonding method is focused on a small-sized sample (1 cm × 1 cm). There is also no relevant report on the tolerance of total dose irradiation of BCB, which is not enough to address and satisfy the strict requirements in the aerospace market. Moreover, the LiNbO_3_ single-crystal thin films prepared by the CIS technology cause a damage layer on the surface of the thin film due to the Frenkel defect, which reduces the single crystal quality and electrical properties of the film. Therefore, we need to explore a new method to achieve the integration of large-area, high-quality LiNbO_3_ single-crystal thin films on Si substrates to meet the stringent requirements of aerospace applications.

Herein, a thin film preparation technology by polyimide (PI) adhesive bonding and chemical mechanical polishing is proposed to achieve the integration of wafer-level LiNbO_3_ single-crystal thin film on Si. PI materials have some advantages in wafer-level bonding for space applications, such as excellently dielectric properties (with a dielectric constant of about 3.4 and a dielectric loss of 10^−3^), good endurance at low temperatures (≥4 K), and strong antiradiation damaging features [24,25,26]. The wafer-level LiNbO_3_ single-crystal was bonded to the Si substrate by polyimide adhesive bonding and plasma activated bonding. The large bonding strength can withstand the stress generated by the harsh chemical-mechanical thinning and polishing. Since PI solvents are relatively difficult to volatilize and by-products are generated during PI curing, it is necessary to prepare high-quality PI films for wafer bonding. Then, the LiNbO_3_ thickness was decreased by chemical mechanical polishing. XRD analysis shows the high crystalline quality of the LiNbO_3_ thin film.

## 2. Experiments

### 2.1. Samples Preparation

Double-side-polished Z-cut LiNbO_3_ wafers in 100 mm and single-side-polished n-type doped [100] Si wafer in 100 mm were used in our study. The wafer thickness was about 500 μm. Prior to spin-coating the PI, the surface cleaning of LiNbO_3_ and Si was carried out according to the RCA cleaning method. First, the wafers were cleaned using the RCA3 solution (H_2_SO_4_:H_2_O_2_ = 3:1) at 150 °C for 15 min; then, the wafers were cleaned by the RCA1 solution (NH_3_H_2_O:H_2_O_2_:H_2_O = 1:3:7) at 150 °C for 5 min. After the above cleaning procedures, all the samples were rinsed using de-ionized water and blow-dried using high-purity nitrogen.

After cleaning, PI (commercial name PI-5100 from Feynman Technology Co, Ltd. Wuxi, Jiangsu Province, China) bonding glue was spin coated on both lithium niobite sample and silicon substrate. In order to obtain a high-quality PI intermediate layer that can be used for bonding, PI thin films under different spin-coating speeds (2000, 3000, 3500, 4000, 5000 rad/min) and different baking conditions (A group: baked on a heat plate at 80 °C for 1 min; B group: baked on a heat plate at 80 °C for 30 min; C group: baked on a hot plate at 80 °C for 30 min and at 150 °C for 30 min; D group: baked on a hot plate at 80 °C for 30 min, at 150 °C for 30 min, and at 250 °C for 30 min) were compared. In order to fully volatilize the solvent and achieve a certain degree of solidification of PI, so that the by-product H_2_O produced in the subsequent bonding process is very small, we used gradient baking in the C and D groups of experiments for soft baking.

**Figure 1:**
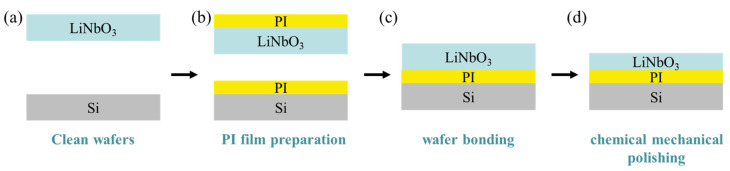
Integration processes of wafer-level LiNbO_3_ single-crystal thin film on silicon: (**a**) wafer cleaning, (**b**) preparation of PI film on LiNbO_3_ and Si, (**c**) LiNbO_3_ substrate and Si substrate bonding, and (**d**) thinning procedure of LiNbO_3_ by CMP.

As a next step, all the samples were activated by using O_2_ plasma sputtering samples surface in the PVA-Tepla-loN40 plasma system. The power, pressure, and activation time of O_2_ plasma are 250 W, 150 mTorr, and 90 s, respectively. The flow rate of O_2_ is 500 sccm. After that, the two wafers were taken out of the plasma chamber and brought into contact at room temperature in air to complete the pre-bonding. After storing for 24 h in air at room temperature to saturate the pre-bonding strength, the pre-bonded wafer pair was annealed at 100 °C for 15 h to complete the bonding. Then, the bonded wafer pairs were bonded to a flat and parallel glass support disc to fit the CMP system. The bonded wafer pairs were thinned by the chemical mechanical thinning system (Logitech LP50, Glasgow, England, UK). In order to obtain an LN surface with a low TTV (total thickness variation) value, we use abrasive grains of different sizes for the grinding process. Finally, LN was polished to about 10 μm by the chemical mechanical polishing system (Logitech LP50, UK). The preparation flow chart of lithium niobate single-crystal film is shown in Figure 1.

### 2.2. Characterization and Measurements

The residual strain of the PI thin film was evaluated by Raman scattering (Renishaw navia) excited by a 532 nm laser. The chemical state of PI thin film was characterized by a Fourier transform infrared spectrometer (FT-IR, Nicolet iS50, Waltham, Massachusetts, USA) and the surface morphology of PI thin film was analyzed by an atomic force microscope (AFM, MFP-3D, Santa Barbara, California, USA). The tensile tester (GOTECH AI-3000S Extensograph, Taiwan, China) was applied to test the bonding strength. In this test, a 1.023 mm-wide bonded sample was pulled at the rate of 200 mm/min, and the average force was calculated to yield the bonding strength. The interfaces of the samples were identified by scanning electron microscopy (SEM, ZEISS SUPRA-55, Oberkochen, Baden-Wurttemberg, Germany). The crystalline quality of LN thin films was examined by X-ray diffraction (XRD, DX-2700B, Dandong, Liaoning Province, China).

## 3. Results and Discussion

**Figure 2:**
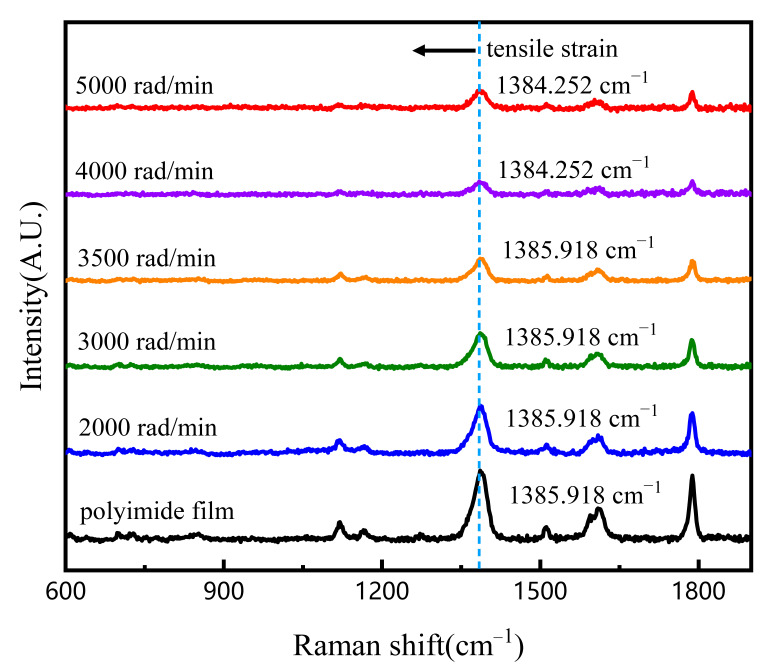
Raman spectra of polyimide film and polyimide films grown on the substrate with different spin-coating conditions.

The thickness of the polyimide intermediate layer is different under different spinning speeds. The stress accumulation of polyimide films of different thicknesses is different during curing, which will affect the subsequent wafer bonding and LiNbO_3_ thin film applications. Excessive stress accumulation will cause bonded samples to fracture during the heat treatment. Due to the residual stress, the materials on both sides of the PI intermediate layer will be subjected to stresses of equal magnitude and opposite directions. The internal lattice of the LiNbO_3_ single-crystal will be distorted and the interplanar spacing will change, which will affect the quality of the LiNbO_3_ single-crystal. We prepared polyimide films of different thicknesses on the substrate by spin coating at 2000 rad/min, 3000 rad/min, 3500 rad/min, 4000 rad/min and 5000 rad/min, and baked them to make them fully cured. The residual strain of the PI film with different spin-coating conditions is characterized by Raman spectroscopy. The Raman scattering spectrum is related to the vibration of solid molecules, and the frequency of peak is equal to the vibration frequency of material atoms [27,28]. If there is stress in the material, some stress-sensitive bands will move and deform [29]. As shown in Figure 2, three vibration peaks were observed in all samples, located at ~1385.918 cm^−1^, ~1609.211 cm^−1^ and 1788.400 cm^−1^. From the Raman spectrum, we can understand that as the thickness of the PI film increases, the Raman peak has a very small frequency shift trend to the lower frequencies, which indicates that there is very weak tensile stress in the film [30]. When the spin-coating speed is 4000 rad/min, the Raman peak has a slight frequency shift, moving from 1385.918 cm^−1^ to 1384.252 cm^−1^, which indicates that an extremely weak tensile stress exists in the PI film during the growth process. In general, the extremely weak tensile stress has a negligible effect on the LiNbO_3_/Si bonded structure.

**Figure 3:**
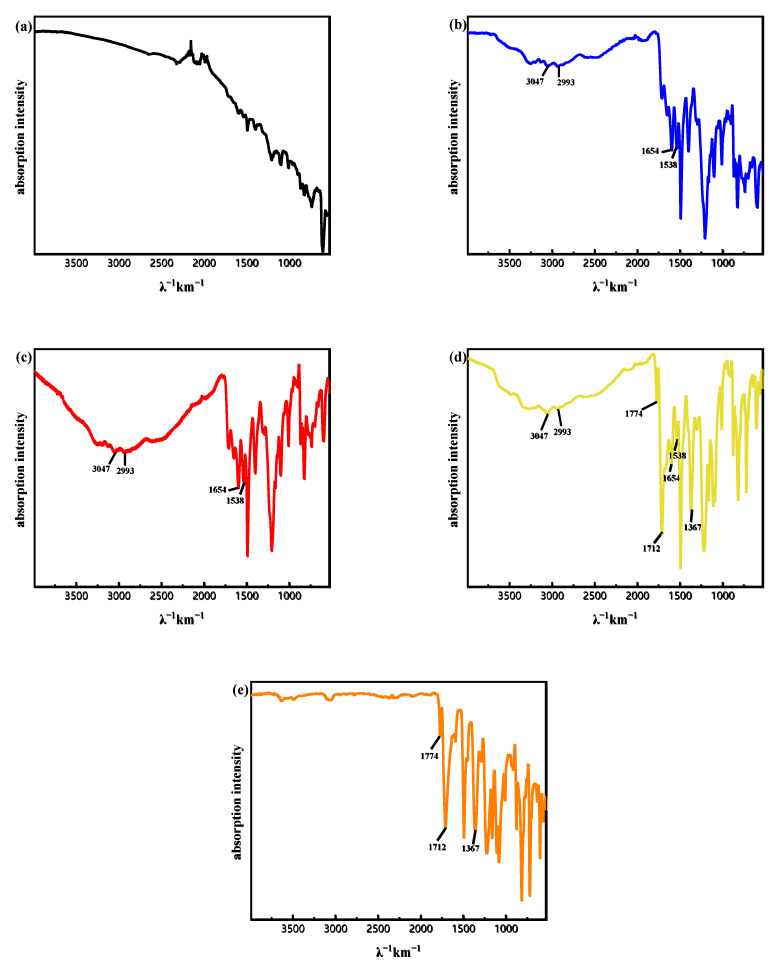
Fourier transform infrared (FT-IR) spectra of PI film after pre-baking at (**a**). 80 °C, 1 min; (**b**). 80 °C, 30 min; (**c**). 80 °C, 30 min; 150 °C, 30 min; (**d**). 80 °C, 30 min; 150 °C, 30 min, 250 °C, 30 min; (**e**). 80 °C, 30 min; 150 °C, 30 min; 250 °C, 2 h.

Polyimide adhesive has excellent adhesion properties. The solute in PI-5100 is polyamide acid, which exists in the organic solvent in the form of a complex. It is difficult to completely remove the solvent, which requires a long period of baking at a higher temperature. The adhesion performance of the PI films gradually decreases during the process of baking to remove the solvent. Moreover, PI films produce by-products during the curing process (polyamide acid forms polyimide through a polycondensation reaction). In order to obtain high-quality PI films that can be used for wafer bonding, PI films pre-baked under different conditions were characterized by FT-IR, as shown in Figure 3. According to Figure 3b, it can be seen that the characteristic peaks of N–H (NH_2_) and O–H (COOH) appear in 2900~3200 cm^−1^, and strong absorption peaks of C=0 (CONH) and C–NH (CONH) appear at 1654 cm^−1^ and 1538 cm^−1^ respectively, indicating that the main component of the film at this time is polyamide acid. With the increase of baking temperature and baking time, the characteristic peaks above disappeared gradually. After baking at 250 °C for 2 h, all the characteristic peaks above disappeared, and asymmetric and symmetrical stretching vibration of C=0 appeared at 1774 cm^−1^ and 1712 cm^−1^, and characteristic peak of C–N appeared at 1367 cm^−1^, indicating that polyamide acid had been completely converted into polyimide. After baking under condition (d), the above peaks exist simultaneously, indicating that the film has achieved a certain degree of curing, but the cross-linking between molecules is not obvious, which lays a good foundation for the success of subsequent bonding.

**Figure 4:**
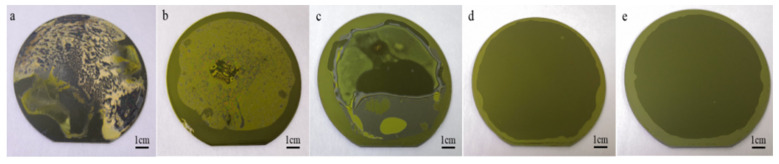
Optical images of glass/Si bonded pairs after pre-baking at (**a**) 80 °C, 1 min; (**b**) 80 °C, 30 min; (**c**) 80 °C, 30 min; 150 °C, 30 min; (**d**) 80 °C, 30 min; 150 °C, 30 min; 250 °C, 30 min; (**e**) 80 °C, 30 min; 150°C, 30 min; 250 °C, 30 min.

Although polyimide has excellent performance, it will produce by-products (H_2_O) during the curing process, which will cause holes in the bonding interface [31]. Therefore, we need to soft bake the polyimide to make the solvent fully volatilize and reach a certain degree of curing [32]. In this way, during the curing process of polyimide, the amount of by-products is very small, so that no voids appear in the bonding interface [33]. In order to research the effect of pre-baking conditions on the wafer bonding, and since the temperature of polyimide curing is above 150 °C, we used 100 mm BF33 wafer and 100 mm Si wafer to perform the aforementioned comparative experiments. Put the bonded wafer pairs into the wafer bonding machine, and heat up to 250 °C for 2 h. As shown in Figure 4a,b, the PI peptizer is not sufficiently volatilized, causing a large number of bubbles and even PI modification at the bonding interface. Due to insufficient pre-curing of PI film, there are a large number of water molecules in the bonding interface, which affects the bonding quality, as shown in Figure 4c. Figure 4d shows that after baking at 80 °C, 150 °C, and 250 °C for 30 min, the peptizer is fully volatilized, the pre-curing degree meets the bonding conditions, and the final uniform, complete, non-porous bonding interface. Based on the above analysis, the polyimide adhesive layer is pre-baking at 80 °C, 150 °C, and 250 °C for 30 min. Figure 4e presents the bonded pairs of LiNbO_3_ wafer and Si wafer after indirect bonding. Due to the transparency of the LiNbO_3_, the resulting bonding interface can be seen by looking through the LiNbO_3_ layer. There are almost no bubbles and voids at the bonded interface, and no cracks can be observed at the interface. The bonding strength of the LiNbO_3_/Si indirect bonded sample is measured to be 11.379 MPa. The high bonding strength can make the bonded samples withstand the stress caused by the harsh mechanical grinding process. Bonded samples maintain high bond strength at low temperatures. Compared with the PI adhesive bonding method proposed in this paper, the bonding method based on the BCB adhesive reported in the published article cannot achieve Si-LiNbO_3_ wafer-level bonding [21,23].

**Figure 5:**
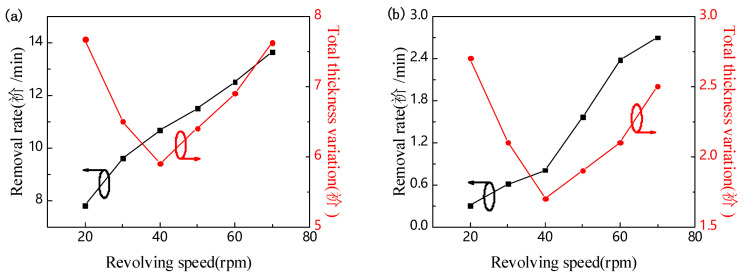
Removal rate and total thickness variation varied with revolving speed of (**a**) processing 1 and (**b**) processing 2.

After the wafer bonding is completed, the LiNbO_3_ single-crystal thin film is prepared by chemical mechanical thinning and polishing. In the lapping 1 process, a Al_2_O_3_ slurry with 20 μm particle size is used to reduce the thickness of the LiNbO_3_ wafer. As shown in Figure 5a, the Al_2_O_3_ slurry with 20 μm particle size has a faster rate of thinning wafer, so thinner LN wafers can be quickly obtained. Black line shows when the speed of the grinding disc was increased from 20 rad/min to 70 rad/min, the removal rate gradually increased from 7.76 μm/min to 13.63 μm/min. Red line shows the TTV value decreases first and then increases with the increase of the rotating speed of the grinding disc, and the minimum value (5.9 μm) is obtained when the rotating speed of the grinding disc is 40 rad/min. The same trend was found in the lapping 2 process. Because the larger particle size of alumina has a greater cutting effect on the LiNbO_3_ wafer, it produces deeper scratches on the surface of the wafer, which can only be repaired by polishing for a long time. The embedded volume of A_2_O_3_ abrasive with 9 μm particle size on the surface of the wafer is smaller than that of A_2_O_3_ abrasive with 20 μm particle size. Therefore, the rate of thinning the wafer by A_2_O_3_ slurry with 9 μm particle size is slower, but the damage on the wafer surface is greatly reduced. When the thickness of the LiNbO_3_ is about 70 μm, the Al_2_O_3_ slurry with 9 μm particle size is used in the lapping 2 process to reduce the thickness of the LiNbO_3_ wafer to about 13 μm. In Figure 5b, black line shows that when the speed of the grinding disc is increased from 20 rad/min to 70 rad/min, the removal rate increases from 0.30 μm/min to 2.70 μm/min. As the speed of the grinding disc increases, red line shows that the TTV value first decreases and then increases. When the speed of the grinding disc is 20 rad/min, the TTV value is 2.70 μm. When the speed of the grinding disc was increased to 40 rad/min, the TTV value reached the minimum, which was 1.7 μm. Subsequently, as the rotation speed of the grinding disc increases, the TTV value also increases. When the speed of the grinding disc is increased to 70 rad/min, the TTV value is 2.5 μm. According to the result of the lapping process, the TTV value will not always decrease as the rotation speed of the grinding disc increases. Too small or too large a rotation speed will cause the fixture rotation speed to be inconsistent with the grinding disc rotation speed. The uneven distribution of the slurry on the bottom of the wafer will result in uneven surface removal rates. Therefore, choosing suitable abrasive grains instead of increasing the speed of the disc to increase the removal rate can minimize the probability of damage.

**Figure 6:**
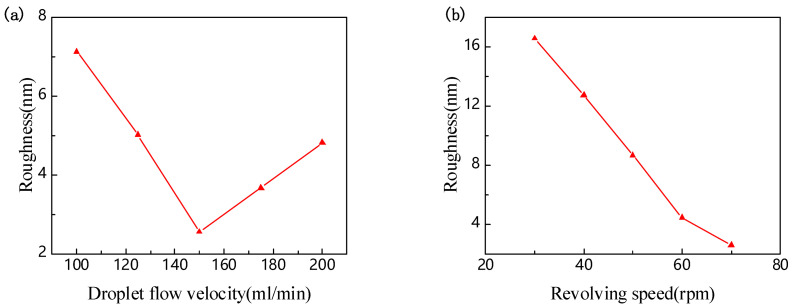
Roughness varied with (**a**) droplet flow velocity and (**b**) revolving speed.

After the lapping process is completed, the damaged layer on the wafer surface needs to be removed by a chemical mechanical polishing process [34,35,36]. Amorphous silica with an abrasive grain size of 50 nm was used for polishing. During the polishing process, the polishing liquid drops on a part of the polishing disc. The centrifugal force caused by the rotation of the polishing disc will evenly distribute the polishing liquid on the surface of the running disc. The polishing liquid is mainly composed of chemical substances and abrasive grains. Chemical substances have a chemical interaction with the polished surface, and abrasive grains have a mechanical (physical) interaction with the polished surface. As shown in Figure 6a, when the rotating speed of the polishing disc is 70 r/min, the wafer surface roughness of the LiNbO_3_ wafer first decreases and then slowly increases with the increase of the slurry flow rate. When the slurry flow rate is 100 mL/min, the wafer surface roughness is 7.13 nm. At this time, the mechanical polishing rate is greater than the chemical corrosion rate. When the slurry flow rate increases, more effective abrasives and solutions participate in the polishing action, resulting in enhanced mechanical grinding and chemical corrosion. However, the chemical corrosion rate is faster than the mechanical grinding rate. When chemical corrosion and mechanical grinding are in balance, the smallest wafer surface roughness can be obtained. When the slurry flow rate is 150 mL/min, the surface roughness reaches the minimum value (2.55 nm). After that, with the increase of the slurry flow rate, the chemical corrosion rate is greater than the mechanical polishing rate. At this time, the wafer surface is corroded severely and the surface roughness increases. When the slurry flow rate is 200 mL/min, the wafer surface roughness is 4.81 nm. The polishing results of LiNbO_3_ at different speeds of the polishing disc are shown in Figure 6b. The slurry flow rate is set to 150 mL/min, and the surface roughness decreases with the increase of the rotation speed. When the rotation speed of the polishing disc is 30 rad/min, the surface roughness is 16.54 nm. As the rotating speed of the polishing disc increases, the mechanical polishing rate increases. When the rotation speed is 70 rad/min and the polishing liquid flow rate is 150 mL/min, the chemical corrosion effect and the mechanical grinding effect are balanced, and the minimum wafer surface roughness (2.55 nm) is obtained.

**Figure 7:**
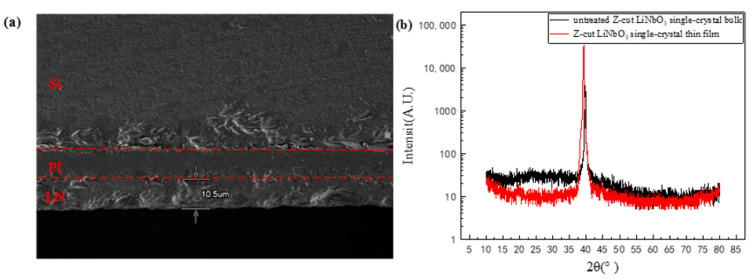
(**a**) Scanning electron microscope (SEM) image and (**b**) X-ray diffraction (XRD) pattern of the LiNbO_3_ single-crystal thin film integrated on Si.

It is important to evaluate the bonding interface. Figure 7a illustrates the SEM cross-sectional view of the integration sample of LiNbO_3_ single-crystal thin film on Si. From top to bottom, Si, PI and LiNbO_3_ single crystal films are in order, and each layer is clearly visible and of uniform thickness. The thickness of LiNbO_3_ single-crystal thin film was measured by SEM, and its thickness was about 10 μm. Moreover, the thickness of the PI film is about 6.16 μm. It can be seen that the bonding interfaces are tightly and clearly visible. The XRD patterns of the LiNbO_3_ thin film is shown in Figure 7b. A Z-cut bulk LN wafer is also measured by XRD as the reference [37,38], and it can be seen that the LiNbO_3_ thin film prepared in this paper is almost consistent with the bulk LN wafer in the XRD pattern. Only one diffraction peak located at 39.28° for the Z-cut LN thin film is observed according to Figure 7b, indicating the single-crystalline structure of the thin film [39]. The FWHMs deduced from the first-order diffraction peak is 0.19° for the Z-cut LiNbO_3_ thin film, demonstrating the highly crystalline quality of the LiNbO_3_ film.

## 4. Conclusions

In this study, an integration technology for wafer-level LiNbO_3_ single-crystal thin film on silicon by polyimide adhesive bonding and chemical mechanical polishing is described. The residual strain and chemical composition of the polyimide film were studied by Raman spectroscopy and FT-IR. The results show that the weak tensile stress has a negligible effect on the LiNbO_3_/Si bonded structure and 80 °C for 30 min, 150 °C for 30 min, and 250 °C for 30 min are the best pre-bake conditions, which can obtain the highest quality PI film that can be used for bonding. The great bonding strength can withstand the stress generated by the harsh chemical-mechanical thinning and polishing, and a large-area, high-quality LiNbO_3_ single-crystal thin film is prepared. After the bonding process, the LiNbO_3_ wafer was lapped by Al_2_O_3_ with abrasive slurry sizes of 20 and 9 μm, respectively, and then polished by an amorphous silica with an abrasive slurry size of 50 nm. Finally, the thickness of the LiNbO_3_ wafer with a 100 mm diameter can be reduced from 500 to 10 μm with almost no cracks. SEM results show that the bonding interface is defect-free and uniform. The LiNbO_3_ single-crystal thin film prepared in this paper meets the requirements of low temperature and other harsh environments for LiNbO3-on-Si devices.

## Data Availability

Data sharing is not applicable to this article.

## References

[1] Janson S., Helvajian H., Breuer K. (1999). MEMS, microengineering and aerospace systems. Proceedings of the 30th Fluid Dynamics Conference.

[2] Li Y., Lu Y., Xie B., Chen J., Wang J., Chen D. (2020). A Micromachined Resonant Differential Pressure Sensor. IEEE Trans. Electron Devices.

[3] Liu H., Pike W.T. (2016). A micromachined angular-acceleration sensor for geophysical applications. Appl. Phys. Lett..

[4] Wu W., Liu Z., Jauregui L.A., Yu Q., Pillai R., Cao H., Bao J., Chen Y.P., Pei S.-S. (2010). Wafer-scale synthesis of graphene by chemical vapor deposition and its application in hydrogen sensing. Sensors Actuators B Chem..

[5] Bourdarie S., Xapsos M. (2008). The Near-Earth Space Radiation Environment. IEEE Trans. Nucl. Sci..

[6] Broomfield G. (1980). The effect of low-fluence neutron irradiation on silver-electroded lead-zirconate-titanate piezoelectric ceramics. J. Nucl. Mater..

[7] Chaiken M.F., Blue T.E. (2018). An Estimation of the Neutron Displacement Damage Cross Section for Ga2O3. IEEE Trans. Nucl. Sci..

[8] Du J., Qiu Y., Zhang J., Huang J., Wu Z., Zhang X., Wang Y., Baldwin J.K., Fu E. (2018). The alleviation of radiation-damage on Nb/MgO film driven by strain gradient in He ion irradiation. Appl. Surf. Sci..

[9] Shea H. (2009). Radiation sensitivity of microelectromechanical system devices. J. Microlithogr. Microfabr. Microsyst..

[10] Drummond E.I. (1987). Resistance of Ti:LiNbO3 devices to ionising radiation. Electron. Lett..

[11] Primak W., Gavin A.P., Anderson T.T., Monahan E. (1977). Stability of Lithium Niobate on Irradiation at Elevated Temperature. Nucl. Technol..

[12] Wong K.-K. (2002). Properties of Lithium Niobate.

[13] Kim K., Choi J.-Y., Kim T., Cho S.-H., Chung H.-J. (2011). A role for graphene in silicon-based semiconductor devices. Nature.

[14] Wahab Y., Zayegh A., Begg R. Silicon implementation of micro pressure sensor. Proceedings of the 2010 International Conference on Electronic Devices, Systems and Applications.

[15] Wu K., Zhang H., Chen Y., Luo Q., Xu K. (2021). All-Silicon Microdisplay Using Efficient Hot-Carrier Electroluminescence in Standard 0.18μm CMOS Technology. IEEE Electron Device Lett..

[16] Almirall A., Oliveri S., Daniau W., Margueron S., Baron T., Boulet P., Ballandras S., Chamaly S., Bartasyte A. (2019). High-Frequency Surface Acoustic Wave Devices Based on Epitaxial Z-LiNbO3 Layers on Sapphire. Appl. Phys. Lett..

[17] Bartasyte A., Plausinaitiene V., Abrutis A., Stanionytė S., Margueron S., Boulet P., Kobata T., Uesu Y., Gleize J. (2013). Identification of LiNbO_3_, LiNb _3_O_8_ and Li_3_ NbO_4_ phases in thin films synthesized with different deposition techniques by means of XRD and Raman spectroscopy. J. Phys. Condens. Matter Inst. Phys. J..

[18] He C., Li X., Qiu D., Chen Y., Lu Y., Cui X. (2021). Nonlinear optical polarization and heterostructure synergistically boosted the built-in electric field of CeF3/LiNbO3 for a higher photocatalytic nitrogen reduction activity. Appl. Surf. Sci..

[19] Shandilya S., Tomar M., Gupta V. (2012). Deposition of stress free c-axis oriented LiNbO3 thin film grown on (002) ZnO coated Si substrate. J. Appl. Phys..

[20] You P., Lu C., Ye W., Hao L., Zhu J., Zhou Y. (2013). Growth of highly near-c-axis oriented ferroelectric LiNbO3 thin films on Si with a ZnO buffer layer. Appl. Phys. Lett..

[21] Bai X., Shuai Y., Gong C., Wu C., Luo W., Böttger R., Zhou S., Zhang W. (2018). Surface modifications of crystal-ion-sliced LiNbO3 thin films by low energy ion irradiations. Appl. Surf. Sci..

[22] Pastureaud T., Solal M., Biasse B., Aspar B., Briot J.-B., Daniau W., Steichen W., Raphael L., Laude V., Laëns A. (2007). High-frequency surface acoustic waves excited on thin-oriented LiNbO3 single-crystal layers transferred onto silicon. IEEE Trans. Ultrason. Ferroelectr. Freq. Control.

[23] Luo W., Luo J., Shuai Y., Zhang K., Wang T., Wu C., Zhang W. (2019). Infrared detector based on crystal ion sliced LiNbO3 single-crystal film with BCB bonding and thermal insulating layer. Microelectron. Eng..

[24] Kaltenbrunner M., Sekitani T., Reeder J., Yokota T., Kuribara K., Tokuhara T., Drack M., Schwödiauer R., Graz I., Bauer-Gogonea S. (2013). An ultra-lightweight design for imperceptible plastic electronics. Nature.

[25] Liaw D.-J., Wang K.-L., Huang S., Lee k.-R., Lai J.-Y., Ha C.-S. (2012). Advanced polyimide materials: Syntheses, physical properties and applications. Prog. Polym. Sci..

[26] Xie J., Xin D., Cao H., Wang C., Zhao Y., Yao L., Ji F., Qiu Y. (2011). Improving carbon fiber adhesion to polyimide with atmospheric pressure plasma treatment. Surf. Coat. Technol..

[27] Inoue K., Asai N., Sameshima T. (1981). Experimental Study of the Hyper-Raman Scattering Due to Raman Inactive Lattice Vibration in SrTiO_3_. J. Phys. Soc. Jpn..

[28] Wang Y., Cong C., Qiu C., Yu T. (2013). Raman Spectroscopy Study of Lattice Vibration and Crystallographic Orientation of Monolayer MoS2under Uniaxial Strain. Small.

[29] Webster S., Batchelder D.N., Smith D.A. (1998). Submicron resolution measurement of stress in silicon by near-field Raman spectroscopy. Appl. Phys. Lett..

[30] Klesse W.M., Scappucci G., Capellini G., Hartmann J.M., Simmons M. (2013). Atomic layer doping of strained Ge-on-insulator thin films with high electron densities. Appl. Phys. Lett..

[31] Duo S., Li M., Zhu M., Zhou Y. (2006). Resistance of polyimide/silica hybrid films to atomic oxygen attack. Surf. Coat. Technol..

[32] Tzu-Chien J.H., Liu Z. (2010). Solvent effect on the curing of polyimide resins. J. Appl. Polym. Sci..

[33] Moon K.H., Chae B., Kim K.S., Lee S.W., Jung Y.M. (2019). Preparation and Characterization of Transparent Polyimide⁻Silica Composite Films Using Polyimide with Carboxylic Acid Groups. Polymers.

[34] Deng H., Hosoya K., Imanishi Y., Endo K., Yamamura K. (2015). Electro-chemical mechanical polishing of single-crystal SiC using CeO2 slurry. Electrochem. Commun..

[35] Ryuzaki D., Hoshi Y., Machii Y., Koyama N., Ashizawa T. (2012). Chemical Mechanical Polishing with Nanocolloidal Ceria Slurry for Low-Damage Planarization of Dielectric Films. Jpn. J. Appl. Phys..

[36] Fukushima A., Fujitani M., Ishikawa K., Numazawa M., Ishi D., Otsubo R., Nagatoshi H., Suzuki H., Yuasa T., Ezoe Y. (2019). Grinding and chemical mechanical polishing process for micropore x-ray optics fabricated with deep reactive ion etching. Appl. Opt..

[37] Stepanenko O., Quillier E., Tronche H., Baldi P., De Micheli M. (2016). Crystallographic and Optical Properties of Z-Cut High Index Soft Proton Exchange (HISoPE) LiNbO3Waveguides. J. Light. Technol..

[38] Rams J., Olivares J., Cabrera J.M. (1997). High-index proton-exchanged waveguides in Z-cut LiNbO3 with undegraded nonlinear optical coefficients. Appl. Phys. Lett..

[39] Bai X., Shuai Y., Gong C., Pan X., Zeng H., Wang T., Luo W., Wu C., Zhang W. (2019). The electrical properties of single-crystalline Z-cut LiNbO3 thin films fabricated by crystal-ion-slicing technique. J. Mater. Sci. Mater. Electron..

